# Elucidating the Kinetics of Expression and Immune Cell Infiltration Resulting from Plasmid Gene Delivery Enhanced by Surface Dermal Electroporation

**DOI:** 10.3390/vaccines1030384

**Published:** 2013-08-28

**Authors:** Janess M. Mendoza, Dinah H. Amante, Gleb Kichaev, Christine L. Knott, William B. Kiosses, Trevor R. F. Smith, Niranjan Y. Sardesai, Kate E. Broderick

**Affiliations:** 1 Inovio Pharmaceuticals Inc., 1787 Sentry Parkway West, Building 18, Suite 400, Blue Bell, PA 19422, USA; 2 The Scripps Research Institute, Core Microscopy Facility, 10550 North Torrey Pines Rd, La Jolla, CA 92037, USA

**Keywords:** intradermal, DNA vaccine, electroporation, kinetics, infiltration

## Abstract

The skin is an attractive tissue for vaccination in a clinical setting due to the accessibility of the target, the ease of monitoring and most importantly the immune competent nature of the dermal tissue. While skin electroporation offers an exciting and novel future methodology for the delivery of DNA vaccines in the clinic, little is known about the actual mechanism of the approach and the elucidation of the resulting immune responses. To further understand the mechanism of this platform, the expression kinetics and localization of a reporter plasmid delivered via a surface dermal electroporation (SEP) device as well as the effect that this treatment would have on the resident immune cells in that tissue was investigated. Initially a time course (day 0 to day 21) of enhanced gene delivery with electroporation (EP) was performed to observe the localization of green fluorescent protein (GFP) expression and the kinetics of its appearance as well as clearance. Using gross imaging, GFP expression was not detected on the surface of the skin until 8 h post treatment. However, histological analysis by fluorescent microscopy revealed GFP positive cells as early as 1 h after plasmid delivery and electroporation. Peak GFP expression was observed at 24 h and the expression was maintained in skin for up to seven days. Using an antibody specific for a keratinocyte cell surface marker, reporter gene positive keratinocytes in the epidermis were identified. H&E staining of treated skin sections demonstrated an influx of monocytes and granulocytes at the EP site starting at 4 h and persisting up to day 14 post treatment. Immunological staining revealed a significant migration of lymphocytic cells to the EP site, congregating around cells expressing the delivered antigen. In conclusion, this study provides insights into the expression kinetics following EP enhanced DNA delivery targeting the dermal space. These findings may have implications in the future to design efficient DNA vaccination strategies for the clinic.

## 1. Introduction

Delivery of vaccines directly to the skin (intradermal, ID) is an attractive immunization strategy in a clinical setting due to a number of dermal-specific features. Skin is the most accessible organ of the human body, the most easily monitored, as well as being a highly immunocompetent target [1,2]. Indeed, the skin contains a resident population of antigen presenting cells (APCs), specifically a large number of Langerhans cells and dermal dendritic cells, so has the potential for increased immunogenicity through direct transfection and presentation. Human skin is the largest organ of the human body and extends to approximately 2 m^2^ in area [1]. The most superficial layer of skin is the stratum corneum (SC) which functions as the primary barrier for this organ. The skin has two broad tissue types, the epidermis and the dermis. The epidermis is a continually keratinizing stratified epithelium. Making up approximately 80%–90% of the cellular population of the epidermis, the predominant cell type is the keratinocyte. These cells play both a structural role as well as being immunologically active. Keratinocytes appear to play a role in initiating cell-mediated immune responses in the skin by cytokine release and adhesion-molecule expression [3]. The other three strata of the epidermis (*S. granulosum*, *S. spinosum*, *S. basale*) all contain keratinocytes at different stages of differentiation as well as the immune Langerhans cells and dermal dendritic cells [1,2]. 

The chief function of the Langerhans cells is to process and present antigens encountered in the epidermal space to naive T cells and to initiate an adaptive immune response [4]. Additional APCs that play a role in the skin immune function and trafficking to regional lymph nodes include veiled cells (resident in the lymphatic system), follicular dendritic cells (resident in the regional lymph nodes), monocytes, macrophages and B cells.

The dermis functions primarily as a scaffold for the epidermis, containing a dense collagen matrix, elastic fibers, and extrafibrillar matrix interspersed with fibroblast cells [1]. It is divided into two layers, the superficial area adjacent to the epidermis called the papillary region and a deep thicker area known as the reticular dermis.

DNA vaccines are a next generation branch of vaccines which offer major benefits over their conventional counterparts [5,6,7,8]. Unlike conventional vaccines, DNA vaccines are gene based expression plasmids that encode specific antigens and do not require isolated virus for production. Unlike inactivated vaccines, DNA vaccines can mimic the immunological effects of infection since they directly transfect the host’s cells. As a result, gene expression occurs via the host’s own machinery, allowing for antigen presentation through both the MHC class I and II pathways. Such gene-based vaccines also offer the ability to develop, optimize and manufacture large doses of vaccine in a cost-effective, rapid manner. Due to the inherent stability of DNA vaccines, they do not require cold-chain storage which is a major logistical issue with some current conventional vaccines and biologics. This has obvious major implications for their distribution and use in developing countries. Most importantly, DNA vaccines are able to generate both a robust antibody and T-cell response [7,8,9,10]. This ability means that DNA vaccination offers a therapeutic solution against many complex diseases such as HIV/AIDS and cancers.

A major obstacle to effective vaccination via gene-based methods is the low efficiency of intracellular delivery. Outside of small rodent models, the delivery of naked DNA through a standard intramuscular (IM) injection is notoriously inefficient. In past studies, this has led to an inability to achieve strong immune responses in large mammals and humans immunized with naked DNA [5,6,7,8]. One physical method to temporarily increase cell permeability is electroporation (EP) and this method has moved to the forefront as the modality of choice for DNA vaccination. 

EP involves the application of brief electrical pulses that result in the creation of temporary aqueous pathways within the lipid bi-layer membranes of mammalian cells. This allows the passage of DNA and other macromolecules through a cell membrane that was previously impermeable to these molecules. As such, EP increases both the uptake and the extent to which drugs and DNA are delivered to the target tissue of interest [11,12,13,14,15]. Historically, EP has been primarily targeted to muscle tissue and currently multiple clinical trials are being conducted using this route of delivery [16,17,18,19,20]. 

By the nature of the target tissue, intramuscular EP is an invasive procedure. In an attempt to improve the vaccination experience from the patients’ perspective, recently there has been a significant move towards developing EP devices that target the dermal region. Since the target tissue of skin is considerably shallower from a depth perspective than skeletal muscle, dermal EP devices can be designed to be much less invasive and even completely non-invasive. This has the important implication from a patient tolerability standpoint of not activating deep nerves and muscles. A typical volume for an IM vaccination would be in the range of 1–2 mL whereas ID vaccination injection volumes are generally limited to no more than 100 µL. This raises obvious issues with dose limitation although the dose sparing ability of skin as a target tissue may mitigate this. To be a clinically relevant platform, it is vital that ID EP would still maintain equivalent efficacy in comparison to IM EP procedures. Historically, it had been proposed that IM EP generated robust cellular responses and ID EP humoral responses. However, the current understanding of the platform implies that ID EP can generate both antibody and cellular responses equally well.

Devices for ID EP can be classified into different categories depending on their mode of action or application. Examples of non-invasive or surface electrodes are devices such as the caliper [21] and plate electrode platforms [22]. Further skin surface electrodes are the MEA (Multi-Electrode Array) [23,24], and the meander electrodes [25]. In general, these platforms make direct contact with the dermal surface without rupturing the stratum cornea of the skin and require relatively high electrical field strength for efficient transfection. Contactless electrodes can consist of a static spark [26] or a corona charge [27,28] and make no direct contact with the patient’s skin. These modalities also have the obvious benefit of a lack of a disposable device component. 

Invasive skin EP device configurations generally consist of an array of multiple needles which penetrate into the skin. Roos *et al*. [29] reported that a device consisting of two parallel rows of 4-needle electrodes (8 in total) using two pulses of 1,125 V/cm and 8 pulses of 275 V/cm field strength resulted in robust immune responses [29,30]. This device was initially assessed in humans to evaluate the safety, effectiveness and relative pain levels of dermal EP [31] and has subsequently been used in several clinical trials to deliver a prostate DNA vaccine (ClinTrials identifier—NCT00859729) and a colorectal cancer DNA vaccine (ClinTrials identifier—NCT00859729).

The CELLECTRA®-3P (Inovio Pharmaceuticals, Blue Bell, PA, USA) is a minimally invasive electroporation device which targets dermal and subcutaneous layers of the skin [32,33,34] with mild EP conditions and minimal tissue damage. The device consists of three-needle (3 mm in length) electrodes forming a triangle microarray to cover the DNA injection site. This depth of penetration treats the entire skin thickness and as such targets the dermal cells in the epidermis, dermis and subdermis. Recently, this device has entered the clinic in two studies (ClinTrials identifier—NCT01403155, NCT01405885) sponsored by Inovio Pharmaceuticals (Blue Bell, PA, USA) addressing the delivery of a multi-strain influenza DNA vaccine. The reduced depth of the minimally invasive electrodes has been shown to significantly increase the tolerability of the procedure compared with IM EP [35]. Since these approaches are considered more tolerable, the ability to deliver prophylactic immunizations becomes a reality using this device platform. 

The electroporation device used for this study is a surface EP device (Inovio Pharmaceuticals, Blue Bell, PA, USA) which features a 4 × 4 array of sharp electrodes that disrupt the stratum corneum, but do not penetrate the epidermis or lower tissue layers [36]. The device design (1.5 mm electrode spacing) and pulse parameters (applied 25 volts) used on this device localize the electrical field to the upper layers of the skin and primarily target the epidermis, rich in APCs, such as Langerhans and dermal dendritic cells.

In previous publications, we had detailed the development of this surface EP device (SEP) and demonstrated the utility of the device to induce plasmid expression in the skin which subsequently resulted in robust immune responses [36]. While this publication outlined the proof-of-concept studies with this delivery modality, we were keen to gain a deeper understanding of the mechanism of action and have the ability to specifically identify transfected cells and peak expression times. To achieve this knowledge, a time course assessment of reporter gene expression on both a gross and cellular level was performed. We were able to observe the morphology of transfected cells as well as assess the kinetics of monocyte and granulocyte infiltration at the treatment site. In addition, we demonstrated that ID EP resulted in migration of lymphocytic cells to the treatment site.

The results from this study provide insights into expression kinetics following EP enhanced DNA delivery targeting the dermal space. These findings may have future implications when designing efficient ID DNA vaccination strategies for the clinic allowing for peak antigen expression to drive the immunization schedule.

## 2. Experimental

### 2.1. Animals

Female Hartley guinea pigs (6 months old) weighing ~350–400 grams were used in this study. The guinea pigs were group housed (4 per cage) with ad libitum access to food and water. Animals were quarantined for two weeks prior to experimentation. All animals were housed and handled according to the standards of the Institutional Animal Care and Use Committee.

### 2.2. Treatment and Tissue Processing

Three guinea pigs were shaved and depilated one day prior to initiating the study for the early time points. All three animals received five separate treatments at each defined time point (1, 2, 4, 6 and 8 h). Therefore there was a total of 15 treatment biopsies generated for each individual time point. To assist with tissue harvesting, animals were treated initially for the 8 h time point and subsequently treated in descending time order. The same experimental set up was applied for the later time points (24 h, 48 h, days 3, 7, 12, 14 and 21) where three animals with five separate treatment sites were used. Again, the treatments were performed in descending order to ensure ease of sacrifice. Each treatment at each time point comprised of a single injection of 50 μg of gWIZ-GFP or gWIZ-RFP (Aldevron LLC, Fargo, ND, USA) in 50 µL of PBS delivered intra-dermally using the Mantoux injection method and immediately followed by electroporation using the surface EP device detailed in the introduction [36]. The Mantoux intradermal injection is a standard clinical technique involving a small gauge needle (usually 29G) inserted parallel to the skin bevel up. The device electrical parameters were three pulses of 100 ms at an applied voltage of 25 volts. The time course spanned 21 days and included early time points of 1, 2, 4, 6 and 8 h. At the relevant times, 8 mm biopsy punches of four of the five treatments from each time point on each animal were taken post-mortem and fixed in 4% paraformaldehyde at 4 °C overnight. The following day, skin biopsies were buffered in a 15% sucrose solution and stored until sectioning at 4 °C. The fifth treatment of each time point on each animal was collected and stored at −20 °C for gross imaging. 

### 2.3. Immunohistochemistry

Biopsies were embedded in OCT Compound and sectioned at a thickness of 15 µm using an OTF Bright Cryostat (Cambridge, UK). Sections from all time points were H&E or DAPI stained and viewed under bright light or fluorescent microscopy. Sections from 1, 2, 4, and 6 h time points were stained with unconjugated primary antibodies against: anti-guinea pig lymphocytes and Langerhans cells (Clone MsGp2, AbD Serotec, Oxford, UK), and anti-keratin 10 (Assay Biotech, Sunnyvale, CA, USA). Sections were then stained with either an anti-mouse Alexa Fluor 555 (MsGp2) or anti-rabbit Alexa Fluor 488 (Keratin) (Life-Technologies, Inc., Grand Island, NY, USA) secondary antibody. An additional stain, Hoechst 33342 (Life Technologies, Inc, Grand Island, NY, USA), was used to visualize nuclei. The slides were then mounted with Fluoromount (Ebioscience, San Diego, CA, USA) and viewed using fluorescent or confocal microscopy.

### 2.4. Imaging

Fluorescent microscopy was carried out using an Olympus BX51 with a Magnafire U-TV1X-2/U-CMAD 3 combo camera for photo acquisition (Olympus, New York, NY, USA). Magnafire software was used to acquire the images.

Confocal Images were obtained with a Zeiss LSM 780 laser scanning confocal Microscope (Carl Zeiss, Inc., Jena, Germany) and processed with Zen 2012 Software (Carl Zeiss Inc., Jena, Germany). Z stacks of images (obtained at 0.3 µm intervals) were collected sequentially using a 63× objective and then maximum projected into single flattened stacks for figures. 

## 3. Results and Discussion

### 3.1. Gene Delivery Enhanced by Dermal Electroporation Induces Sustained Expression on the Surface of the Skin

**Figure 1:**
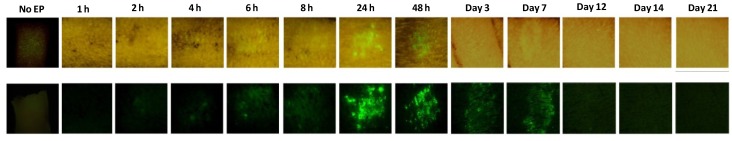
Gene delivery enhanced by dermal electroporation induces sustained expression on the surface of the skin. Time course (1 h post treatment to day 21) of green fluorescent protein (GFP) expression after intradermal (ID) plasmid administration followed by surface electroporation (SEP) in guinea pig skin visualized under natural (top panel) and fluorescent (lower panel) light. An untreated control is also shown. Photos are representative examples of multiple treatments.

To assess the expression kinetics resulting from gene delivery with a dermal EP device, a plasmid expressing GFP was injected into guinea pig skin at defined time points. Surface EP (SEP) was immediately performed after each injection. The animals were sacrificed post treatments and the skin excised and visualized under a fluorescent microscope (Figure 1). GFP expression appeared at 6 h on the surface of the skin and persisted for seven days. The peak expression was observed at 24 h. During the peak times (24*–*72 h), the expression was robust and matched in size the diameter of the injection bubble (approximately 5 mm). Within the gross localization pattern of GFP, smaller islands of expression were also noted which coincided in shape and spacing with the direct contact the electrodes make with the skin. The biopsies shown in the figure are representative examples of those seen in multiple treatments on multiple animals.

### 3.2. Histological Analysis Reveals GFP Expression Appearing after One Hour

**Figure 2:**
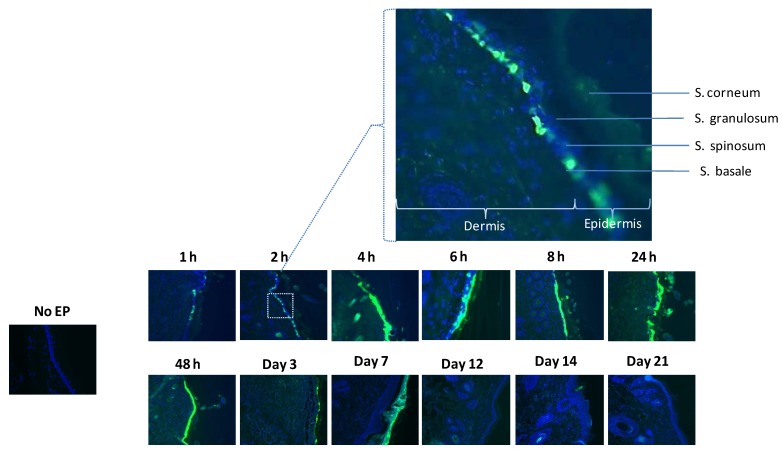
Histological analysis reveals rapid GFP expression. Histological analysis of GFP expression in a time course (1-hour post treatment to day 21) after ID plasmid administration followed by EP with SEP in guinea pig skin. Skin biopsies were removed, cryosectioned, DAPI stained and visualized under fluorescence microscopy (10×). An injection only control is also shown. Photos are representative examples of multiple treatments. A region of the 2-hour time point section image is enlarged to allow the annotation of skin structures.

While the skin surface localization patterns delineate the global expression trends, skin is a squamous epithelial tissue so it was possible that cells below the observable surface (and so not apparent on the surface view) were also being transfected. Through the natural migration of cells in the epidermis, such cells would only later move to the skin surface and so give a falsely slow dynamic. To investigate this further, biopsies were taken of the time points from the original study and fixed sections prepared. Under a fluorescent microscope, sections were observed and scored for GFP positive cells (Figure 2). One hour post treatment, positive cells were identified in skin sections. These cells appeared to be closer to the basement membrane and were likely to be located in the stratum basale level of the epidermis and the mid to upper epidermis (stratum granulosum). As the time course progressed, the transfected cells migrated upwards to the surface of the skin where between day 5 and day 7 the majority of the signal appeared to reside in the stratum corneum. The seemingly brighter GFP signal at day 7 over day 3 is a function of the change in morphology of the reporter gene positive calls as they flatten out in the SC. Post day 7, the GFP positive cells appeared to slough off which is the nature of that barrier layer of the skin. To allow annotation of the structural elements in the skin sections, an enlarged view of the 2 h section is included where the sub-structures and stratum of the skin are noted. In keeping with the shallow electrical field generated by this EP device, all the transfected cells were localized in the epidermis of the skin sections.

**Figure 3:**
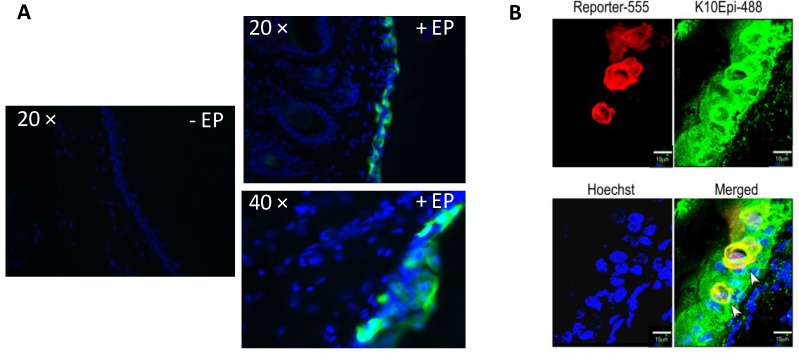
Histological analysis reveals reporter gene expression localized to cells in the epidermis. Histological analysis of GFP and red fluorescent protein (RFP) expression after ID plasmid administration followed by SEP in guinea pig skin. (**A**) GFP treated skin biopsies were removed 4 h post treatment, cryosectioned, DAPI stained and visualized using fluorescence microscopy (20× and 40×). An injection only control (no EP) is also shown; (**B**) RFP treated skin biopsies were removed, cryosectioned, stained with an antibody against K10 (a keratinocyte cell surface marker), Hoechst stained and visualized using confocal imaging.

Magnified DAPI stained images from the 4 h time point plus and minus electroporation (Figure 3A) demonstrate the morphology of the transfected cells as well as the enhancing effect of electroporation. Using a keratinocyte specific antibody, we were able to identify reporter gene positive cells (in this case, red fluorescent protein (RFP)) which also co-stained for K10, the keratinocyte marker (Figure 3B) visualized with an Alexa 488 secondary antibody. In addition to the positive antibody staining, the morphology of the cells depicted through the reporter gene expression is indicative of keratinocytes.

### 3.3. Infiltration at the Electroporation Treatment Site is Fast and Persistent

**Figure 4:**
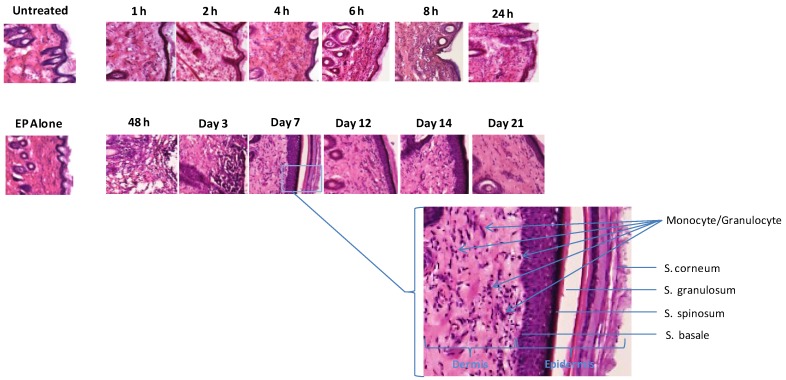
Rapid and persistent monocyte and granulocyte infiltration is detected at the treatment site following EP-enhanced plasmid delivery. Hematoxylin and eosin (H&E) stained histological analysis of skin sections following ID plasmid administration and EP with SEP in guinea pig skin in a time course. Skin biopsies were removed, cryosectioned, H&E stained and visualized under standard light microscopy (10×). An untreated control (no DNA, no EP) and EP alone post 1 h are also shown. A region of the day 7 time point section is enlarged to indicate skin structures and infiltration of monocyte and granulocyte.

The biopsied sections from Figure 2 were additionally stained with H&E to observe the dynamics of infiltration at the site following EP treatment (Figure 4). Unlike infiltration in the muscle following EP, which is generally a relatively slow process, monocytes and granulocytes were observed migrating to the skin treatment site within 4 h. The inflammatory context persisted for 14 days and appeared resolved at day 21. No evidence of necrosis or localized tissue damage as a result of the EP was observed at any time point. To allow closer analysis of the infiltration and annotation of the structural elements in the skin sections, an enlarged view of the day 7 section is included where the sub-structures and stratum of the skin are noted.

### 3.4. Pronounced Migration of Lymphocytic Cells Is Observed at the Site of Gene Transfer Enhanced by Electroporation

**Figure 5:**
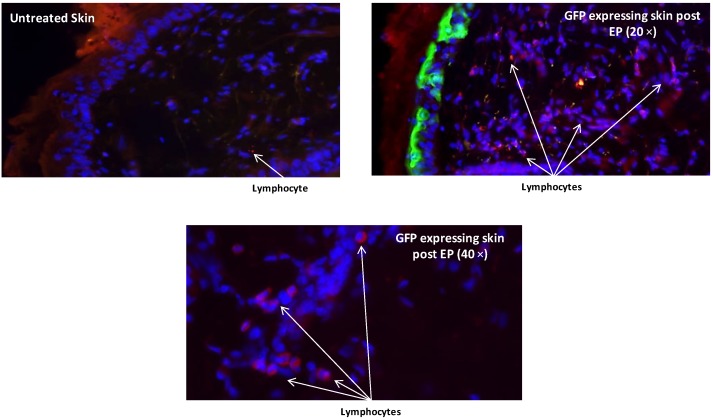
Histological analysis reveals infiltration of lymphocytes at the EP treatment site. Guinea pig skin sections were immunohistochemically stained with an antibody recognizing lymphocytes and Langerhans cells (Tetramethylrhodamine isothiocyanate (TRITC) secondary) after ID GFP plasmid administration followed by EP with SEP. Skin biopsies were removed, cryosectioned, antibody and 4',6-diamidino-2-phenylindole (DAPI) stained and visualized under fluorescence microscopy at 20× and 40×. An untreated control (no DNA, no EP) is also shown.

The major APC in the epidermis is the Langerhans cell. Using an antibody against Langerhans cells and lymphocytes, we immunostained skin sections to observe the dynamics of these migratory cells following EP. Although increased numbers of lymphocytic cells were observed following DNA injection alone and EP alone (data not shown), a significant increase in cell numbers was observed following EP enhanced delivery of plasmid expressing GFP at 6 h post treatment (Figure 5). Through analysis of multiple GFP treated sections, lymphocytic cells could be seen migrating towards and congregating in the areas of the skin biopsy where the transfected GFP expressing cells appeared.

## 4. Conclusions

Intradermal electroporation is a platform technology which offers a solution to the tolerable delivery of DNA vaccines in the clinic for prophylactic immunization. Many groups have shown the utility of this technology in a range of animal models in preclinical studies as well as recently in human skin in the clinic. Multiple modalities of ID EP devices exist, ranging from contactless to fully penetrating. Since each device has varying modes of action, each will target different compartments in the skin. This will result in the transfection of different resident cell populations and as such, have the capacity to elicit varying immune responses. While a wealth of published literature demonstrates the ability of the platform to elicit robust immune responses in a spectrum of animal models and in the clinic [29,30,31], less is understood about the mechanism of action of dermal EP, especially related to the resulting expression kinetics. Incidences of inflammation at the treatment site following EP has been investigated in guinea pig skin [23] and expression of reporter gene plasmids in skin has been used as a marker by multiple groups. However, these are generally observations at a single time point. Here we investigated the expression of a reporter gene construct following electroporation with a surface EP device that specifically targets the epidermis over a defined time course. This study allowed us insight into peak expression times, duration of expression, and kinetics of infiltration and induced migration of APCs.

An elegant study by Roos *et al.* 2009 [29] investigated the functional properties of invasive EP enhanced intradermal DNA delivery in a mouse model. This group evaluated the kinetics of luciferase transgene expression following DNA injection. Additionally, they identified the location of transfected cells in the skin, the effect on the local tissue environment and the persistence of DNA molecules at the injection site. The work detailed here builds on the Roos study by investigating GFP expression via surface EP in a guinea pig model, histologically identifying transfected cells as well as the kinetics of infiltration at the treatment site.

The animal model of choice for many dermatological applications is the guinea pig. This is primarily due to the similarity in skin physiology between these rodents and humans. All studies detailed here were carried out in the Hartley guinea pigs model. A significant difference between the guinea pig model and human skin is the turnover time of cells in the epidermis. The guinea pig has a faster turnover—approximately 2–3 times faster than human skin cell turnover—but is slower than mouse skin cell turnover—approximately seven days. 

This study allowed us to assess the resulting GFP localization in skin following EP enhanced delivery with a surface device. Here we delivered a 50 µL injection of 50 µg reporter gene plasmid by standard Mantoux ID injection means. While a clear benefit to skin vaccination is the ability to dose-spare, we chose this high dose of reporter plasmid to ensure maximal expression. The resulting injection bubble is approximately 4.5 mm in diameter and so fits appropriately under the electrode array of the SEP device. At the peak expression time point (24 h), the GFP expression pattern corresponds well with the bubble size and array contact. It is also possible to observe small islands of transfection. We believe these islands correspond directly to the contact made between the electrode and the skin.

The GFP expression following EP with this device was observed as early as 1 h (microscopically) and persisted through day 7. This timing coincides well with the turnover of cells in the epidermis in guinea pigs. The turnover of cells in humans is considerably longer, more in the range of weeks than days. Analysis of the skin sections suggests that we are directly transfecting cells both in the stratum basale and in the mid to upper epidermis (stratum granulosum) which over the next seven days differentiate as they move towards the upper barrier layer of the stratum corneum. Once trapped in this non-viable but biologically active layer, the GFP disappears as the skin upper layer is sloughed off. Due to the distinct electrode spacing and the low applied voltage of the SEP device, only observing transfection of cells in the epidermis makes sense. The electric field generated by such a modality would be shallow and not penetrate further into the dermis. We believe that this feature of the device will lead to a highly tolerable platform since deep nerves and skeletal muscle will not be activated during the procedure. 

Higher magnification depictions of the GFP/RFP positive cells in the epidermis reveal distinct cellular morphologies similar to a keratinocyte cell. We confirmed this by additionally staining for a keratinocyte cell surface marker (K10) and observing both antibody positive cells (using an Alexa 488 secondary antibody) and reporter gene positive cells. This is an intuitive finding since keratinocytes make up between 80%–90% of the epidermis cellular population. Since the applied voltage parameter of this device is 25 volts, and the electrode spacing is 1.5 mm, the resulting electrical field is mild and shallow. As such, the finding that there was no obvious cellular damage or treatment associated necrosis was unsurprising. In a previous publication [37], we demonstrated that the lack of skin damage at these low voltages did not compromise the resulting immune responses. 

Following EP in the muscle, infiltration at the site is not observed until 4 days post treatment [38]. In this H&E skin study; we observed significant monocyte/granulocyte trafficking to the treatment site at the 4 h time point. Clearly there are significant differences between skin and muscle as target tissues but this finding seeks to highlight the benefits of intradermal vaccinations from the perspective of rapid dynamics. Interestingly, increased infiltration is still observed in EP-treated skin 14 days post procedure, significantly longer than the persistence of reporter gene expression (seven days). It is possible that a low number of cells are still expressing the antigen at these later time-points but are below our levels of imaging detection. 

Antibody staining for lymphocytes/Langerhans cells demonstrated significant increases in detected cells following EP-enhanced plasmid delivery over untreated skin. The majority of positive cells were detected in the dermis region. It is possible that increased numbers of cells would also be detected in the epidermis (alongside the reported gene expression) however more sophisticated imaging equipment may be required to observe this. Ongoing studies are currently underway to further assess the dynamics of this infiltration.

When designing clinical protocols involving plasmid transfer, an understanding of the optimal operating parameters of the vaccine delivery device is crucial. The information gained from this study might allow us to design optimal prime/boost regimes from a timing perspective, taking into account expression kinetics and trafficking of immune sensing cells to the treatment site.

## References

[1] Tobin D.J. (2006). Biochemistry of human skin—Our brain on the outside. Chem. Soc. Rev..

[2] Toebak M.J., Gibbs S., Bruynzeel D.P., Scheper R.J., Rustemeyer T. (2009). Dendritic cells: Biology of the skin. Contact Derm..

[3] Nickoloff B.J., Turka L.A., Mitra R.S., Nestle F.O. (1995). Direct and indirect control of T-cell activation by keratinocytes. J. Invest. Dermatol..

[4] Romani N., Holzmann S., Tripp C.H., Koch F., Stoitzner P. (2003). Langerhans cells—Dendritic cells of the epidermis. APMIS.

[5] Weiner D.B. (2008). DNA vaccines: Crossing a line in the sand. Introduction to special issue. Vaccine.

[6] Donnelly J.J., Ulmer J.B., Liu M.A. (1997). DNA vaccines. Life Sci..

[7] Andre S., Seed B., Eberle J., Schraut W., Bultmann A., Haas J. (1998). Increased immune response elicited by DNA vaccination with a synthetic gp120 sequence with optimized codon usage. J. Virol..

[8] Sardesai N.Y., Weiner D.B. (2011). Electroporation delivery of DNA vaccines: Prospects for success. Curr. Opin. Immunol..

[9] Martinon F., Kaldma K., Sikut R., Culina S., Romain G., Tuomela M., Adojaan M., Mannik A., Toots U., Kivisild T. (2009). Persistent immune responses induced by a human immunodeficiency virus DNA vaccine delivered in association with electroporation in the skin of nonhuman primates. Hum. Gene Ther..

[10] Song J.M., Kim Y.C., Lipatov A.S., Pearton M., Davis C.T., Yoo D.G., Park K.M., Chen L.M., Quan F.S., Birchall J.C. (2010). Microneedle delivery of H5N1 influenza virus-like particles to the skin induces long-lasting B- and T-cell responses in mice. Clin. Vaccine Immunol..

[11] Mathiesen I. (1999). Electropermeabilization of skeletal muscle enhances gene transfer *in vivo*. Gene Ther..

[12] Otten G., Schaefer M., Doe B., Liu H., Srivastava I., zur Megede J., O’Hagan D., Donnelly J., Widera G., Rabussay D. (2004). Enhancement of DNA vaccine potency in rhesus macaques by electroporation. Vaccine.

[13] Otten G.R., Schaefer M., Doe B., Liu H., Megede J.Z., Donnelly J., Rabussay D., Barnett S., Ulmer J.B. (2006). Potent immunogenicity of an HIV-1 gag-pol fusion DNA vaccine delivered by *in vivo* electroporation. Vaccine.

[14] Prud’homme G.J., Draghia-Akli R., Wang Q. (2007). Plasmid-based gene therapy of diabetes mellitus. Gene Ther..

[15] Widera G., Austin M., Rabussay D., Goldbeck C., Barnett S.W., Chen M., Leung L., Otten G.R., Thudium K., Selby M.J. (2000). Increased DNA vaccine delivery and immunogenicity by electroporation *in vivo*. J. Immunol..

[16] Kopycinski J., Cheeseman H., Ashraf A., Gill D., Hayes P., Hannaman D., Gilmour J., Cox J.H., Vasan S. (2012). A DNA-based candidate hiv vaccine delivered via *in vivo* electroporation induces CD4 responses toward the α4β7-binding V2 loop of HIV gp120 in healthy volunteers. Clin. Vaccine Immunol..

[17] Diaz C.M., Chiappori A., Aurisicchio L., Bagchi A., Clark J., Dubey S., Fridman A., Fabregas J.C., Marshall J., Scarselli E. (2013). Phase 1 studies of the safety and immunogenicity of electroporated HER2/CEA DNA vaccine followed by adenoviral boost immunization in patients with solid tumors. J. Transl. Med..

[18] Chudley L., McCann K., Mander A., Tjelle T., Campos-Perez J., Godeseth R., Creak A., Dobbyn J., Johnson B., Bass P. (2012). DNA fusion-gene vaccination in patients with prostate cancer induces high-frequency CD8+ T-cell responses and increases PSA doubling time. Cancer Immunol. Immunother..

[19] Vasan S., Hurley A., Schlesinger S.J., Hannaman D., Gardiner D.F., Dugin D.P., Boente-Carrera M., Vittorino R., Caskey M., Andersen J. (2011). *In vivo* electroporation enhances the immunogenicity of an HIV-1 DNA vaccine candidate in healthy volunteers. PLoS One.

[20] Bagarazzi M.L., Yan J., Morrow M.P., Shen X., Parker R.L., Lee J.C., Giffear M., Pankhong P., Khan A.S., Broderick K.E. (2012). Immunotherapy against HPV16/18 generates potent Th1 and cytotoxic cellular immune responses. Sci. Transl. Med..

[21] Zhang L., Li L., Hoffmann G.A., Hoffman R.M. (1996). Depth-targeted efficient gene delivery and expression in the skin by pulsed electric fields: An approach to gene therapy of skin aging and other diseases. Biochem. Biophys. Res. Commun..

[22] Heller L.C., Jaroszeski M.J., Coppola D., McCray A.N., Hickey J., Heller R. (2007). Optimization of cutaneous electrically mediated plasmid DNA delivery using novel electrode. Gene Ther..

[23] Donate A., Coppola D., Cruz Y., Heller R. (2011). Evaluation of a novel non-penetrating electrode for use in DNA vaccination. PLoS One.

[24] Heller R., Cruz Y., Heller L.C., Gilbert R.A., Jaroszeski M.J. (2010). Electrically mediated delivery of plasmid DNA to the skin, using a multielectrode array. Hum. Gene Ther..

[25] Zhang L., Nolan E., Kreitschitz S., Rabussay D.P. (2002). Enhanced delivery of naked DNA to the skin by non-invasive *in vivo* electroporation. Biochim. Biophys. Acta.

[26] Broderick K.E., Kardos T., McCoy J.R., Fons M.P., Kemmerrer S., Sardesai N.Y. (2011). Piezoelectric permeabilization of mammalian dermal tissue for *in vivo* DNA delivery leads to enhanced protein expression and increased immunogenicity. Hum. Vaccin..

[27] Connolly R.J., Chapman T., Hoff A.M., Kutzler M.A., Jaroszeski M.J., Ugen K.E. (2012). Non-contacthelium-based plasma for delivery of DNA vaccines. Enhancement of humoral and cellular immune responses. Hum. Vaccin. Immunother..

[28] Connolly R.J., Rey J.I., Lambert V.M., Wegerif G., Jaroszeski M.J., Ugen K.E. (2011). Enhancement of antigen specific humoral immune responses after delivery of a DNA plasmid based vaccine through a contact-independent helium plasma. Vaccine.

[29] Roos A.K., Eriksson F., Timmons J.A., Gerhardt J., Nyman U., Gudmundsdotter L., Brave A., Wahren B., Pisa P. (2009). Skin electroporation: Effects on transgene expression, DNA persistence and local tissue environment. PLoS One.

[30] Roos A.K., Moreno S., Leder C., Pavlenko M., King A., Pisa P. (2006). Enhancement of cellular immune response to a prostate cancer DNA vaccine by intradermal electroporation. Mol. Ther..

[31] El-Kamary S.S., Billington M., Deitz S., Colby E., Rhinehart H., Wu Y., Blackwelder W., Edelman R., Lee A., King A. (2012). Safety and tolerability of the Easy Vax clinical epidermal electroporation system in healthy adults. Mol. Ther..

[32] Hirao L.A., Wu L., Khan A.S., Satishchandran A., Draghia-Akli R., Weiner D.B. (2008). Intradermal/subcutaneous immunization by electroporation improves plasmid vaccine delivery and potency in pigs and rhesus macaques. Vaccine.

[33] Laddy D.J., Yan J., Khan A.S., Andersen H., Cohn A., Greenhouse J., Lewis M., Manischewitz J., King L.R., Golding H. (2009). Electroporation of synthetic DNA antigens offers protection in nonhuman primates challenged with highly pathogenic avian influenza virus. J. Virol..

[34] Hirao L.A., Draghia-Akli R., Prigge J.T., Yang M., Satishchandran A., Wu L., Hammarlund E., Khan A.S., Babas T., Rhodes L. (2010). Multivalent smallpox DNA vaccine delivered by intradermal electroporation drives protective immunity in nonhuman primates against lethal monkeypox challenge. J. Infect. Dis..

[35] Diehl M.C., Lee J.C., Daniels S.E., Tebas P., Khan A., Giffear M., Sardesai N.Y., Bagarazzi M.L. (2013). Tolerability of intramuscular and intradermal delivery by cellectra® adaptive constant current electroporation device in healthy volunteers. Hum. Vaccin. Immunother..

[36] Broderick K.E., Shen X., Soderholm J., Lin F., McCoy J., Khan A.S., Yan J., Morrow M.P., Patel A., Kobinger G.P. (2011). Prototype development and preclinical immunogenicity analysis of a novel minimally invasive electroporation device. Gene Ther..

[37] Lin F., Shen X., Kichaev G., Mendoza J.M., Yang M., Armendi P., Yan J., Kobinger G.P., Bello A., Khan A.S. (2012). Optimization of electroporation-enhanced intradermal delivery of DNA vaccine using a minimally invasive surface device. Hum. Gene Ther. Methods.

[38] Gronevik E., von Steyern F.V., Kalhovde J.M., Tjelle T.E., Mathiesen I. (2005). Gene expression and immune response kinetics using electroporation-mediated DNA delivery to muscle. J. Gene Med..

